# Transgenic Mouse Model Harboring the Transcriptional Fusion *Ccl20*-Luciferase as a Novel Reporter of Pro-Inflammatory Response

**DOI:** 10.1371/journal.pone.0078447

**Published:** 2013-11-12

**Authors:** Martina Crispo, Laurye Van Maele, Julien Tabareau, Delphine Cayet, Agustina Errea, Ana María Ferreira, Martin Rumbo, Jean Claude Sirard

**Affiliations:** 1 Unidad de Animales Transgénicos y de Experimentación – Institut Pasteur de Montevideo, Montevideo, Uruguay; 2 Institut Pasteur de Lille, Centre d’Infection et d’Immunité de Lille, F-59000 Lille, Region Nord-Pas de Calais, France; 3 Institut National de la Santé et de la Recherche Médicale, U1019, F-59000 Lille, Region Nord-Pas de Calais France; 4 Centre National de la Recherche Scientifique, UMR 8204, F-59000 Lille, Region Nord-Pas de Calais France; 5 Univ Lille Nord de France, F-59000 Lille, Region Nord-Pas de Calais, France; 6 Laboratorio de Investigaciones del Sistema Inmune (LISIN) – National University of La Plata, Provincia de Buenos Aires, Argentina; 7 Catedra de Inmunologia, Facultad de Ciencias/Facultad de Quimica, Universidad de la República, Montevideo, Uruguay; Université Libre de Bruxelles, Belgium

## Abstract

The chemokine CCL20, the unique ligand of CCR6 functions as an attractant of immune cells. Expression of CCL20 is induced by Toll-like Receptor (TLR) signaling or proinflammatory cytokine stimulation. However CCL20 is also constitutively produced at specific epithelial sites of mucosa. This expression profile is achieved by transcriptional regulation. In the present work we characterized regulatory features of mouse *Ccl20* gene. Transcriptional fusions between the mouse *Ccl20* promoter and the firefly luciferase (*luc*) encoding gene were constructed and assessed in *in vitro* and *in vivo* assays. We found that liver CCL20 expression and luciferase activity were upregulated by systemic administration of the TLR5 agonist flagellin. Using shRNA and dominant negative form specific for mouse TLR5, we showed that this expression was controlled by TLR5. To address *in situ* the regulation of gene activity, a transgenic mouse line harboring a functional *Ccl20-luc* fusion was generated. The luciferase expression was highly concordant with *Ccl20* expression in different tissues. Our data indicate that the transgenic mouse model can be used to monitor activation of innate response *in vivo*.

## Introduction

Chemokines are small proteins that play a major role in the chemotactic movement of cells along the body. They are critical for the proper positioning of immune cells and the development of immune responses. Thus, the expression of chemokines is highly regulated [1]. Chemokines can be divided into constitutive and inducible chemokines with regards to their function. Constitutively expressed chemokines contribute at steady state to positioning of immune cells in lymphoid and non lymphoid organs [2]. Inducible chemokines are swiftly and transiently expressed upon stimulation by external factors and promotes the migration of immune cells to stimulation sites [3]. In pro-inflammatory immune responses triggered during infection, inducible chemokines orchestrate the entry of leukocytes such as neutrophils or inflammatory monocytes into the site of injury.

Some chemokines may participate in both homeostatic and alarm-driven functions. The chemokine CCL20 (or LARC for liver- and activation-regulated chemokine) is constitutively expressed in specific sites such as follicle-associated epithelium of Peyer’s patches, isolated lymphoid follicles and tonsils. In addition, the production of CCL20 is strongly upregulated by various danger stimuli in different organs such as liver, intestine or lung and also mucosal sites [4]–[7]. The gene coding for CCL20 represents a stereotypical signature of innate response to infection as determined by meta-analysis of microarray data [8], [9]. CCR6, the unique receptor of CCL20, is expressed by B cells, activated T cells (particularly Th17), regulatory T cells, immature DC or innate lymphoid cells [10]–[14]. How CCL20 participates in constitutive and inducible arms of immunity is supported by the analysis of the promoters from human (*CCL20*) and mouse (*Ccl20*) genes [15]–[18]. Conserved motifs specific for binding of transcription regulators were identified, including the canonical NF-κB and the Ets (E-twenty six) family binding sites [15], [17], [19] The promoters of CCL20 encoding genes are interesting candidates for the establishment of reporter systems. We have previously characterized the *CCL20* human promoter gene and generated stable reporter human intestinal cell lines [19], [20] to investigate how microbial products regulate the transcriptional activity of the gene [21], [22].

The flagellin family, the major constituent of bacteria flagella, forms the unique agonist of Toll-like receptor 5 (TLR5) signaling [23]. Whereas there are differences in human and mouse TLR5 sequences, the specific recognition of flagellins seems to require similar TLR5 determinants [24]. Remarkably, a stop mutation of human TLR5 (1–392) acts as a dominant negative mutant on TLR5 signaling [25]. MyD88 is an essential adaptor molecule for TLR5 signaling since responses are totally abolished in MyD88-knockout animals [26], [27]. TLR5 stimulates transcription of pro-inflammatory genes dependent on NF-κB and MAPKs. Interestingly, flagellins signal very efficiently in both hematopoietic cells including dendritic cells, and stromal cells, especially intestinal epithelial cells or liver cells [6], [28], [29]. Thus, flagellin is a potent activator of CCL20 in human and mouse systems [30], thereby constituting an appropriate way to address regulation of gene coding CCL20.

In this work, we characterized regulatory features of the mouse *Ccl20* gene and generated a mouse reporter system. To this aim, transcriptional fusions between the *Ccl20* promoter and the firefly luciferase (*luc*) encoding gene were constructed and assessed in *in vitro* and *in vivo* assays. Flagellin was used to evaluate the function of promoter and the specificity of regulation with shRNA against *Tlr5* and a dominant form of mouse TLR5 (TLR5_393stop_). A functional *Ccl20-luc* fusion was finally integrated in a mouse line to address *in situ* the regulation of gene activity. We showed that this model can be used to monitor activation of innate response *in vivo*, with maximal expression in the liver.

## Materials and Methods

### Animals

BALB/cJ, C57BL/6J and the transgenic line U68 harboring the *Ccl20-luc-IRES-EYFP* construct were bred at the SPF animal facility of the Transgenic and Experimental Animal Unit of Institut Pasteur de Montevideo or Institut Pasteur de Lille (agreement#A59-350009). *NFκB-RE-luc* mice were purchased from Taconic (Germantown, NY, USA). Experimental procedures complied with international FELASA guidelines, national regulations and ethical guidelines (French protocol CEEA 052011 and Uruguayan Animal Care Committee) and all experimental procedures involving animals were approved by the corresponding Institutional Animal Care Committees of either Institut Pasteur de Lille or Institute Pasteur de Montevideo.

### Plasmid Constructs

**Figure 1:**
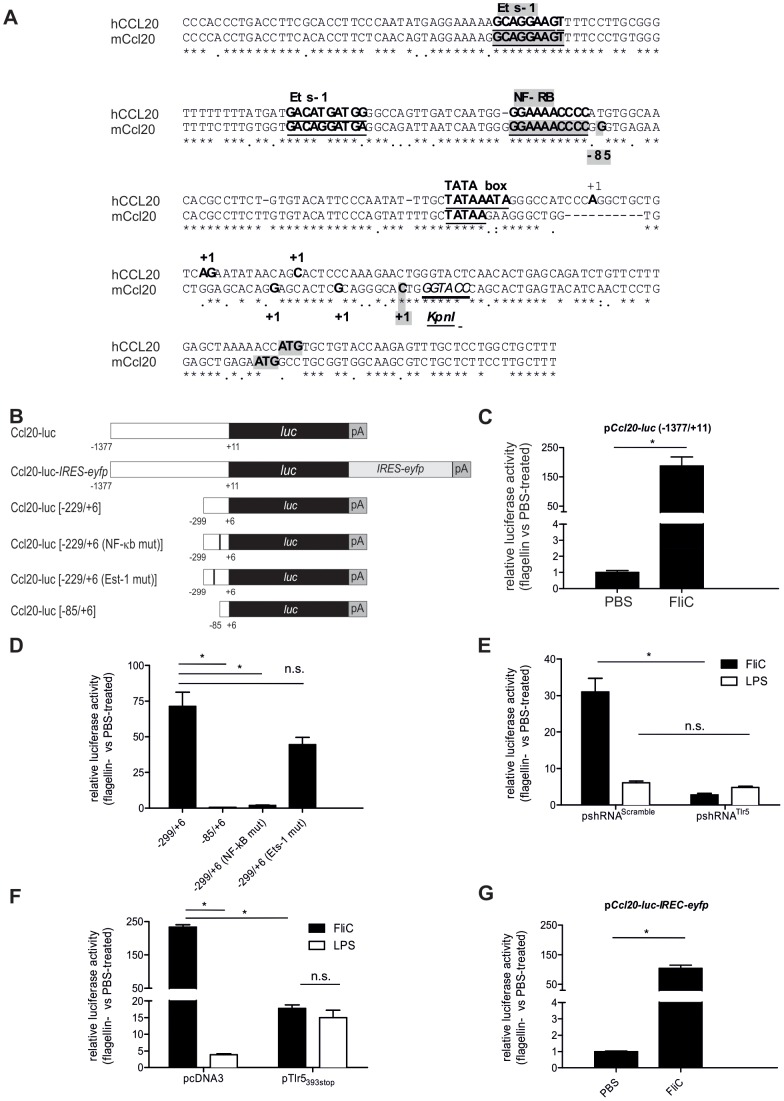
Functional characterization of *Ccl20-luc* transcriptional fusions. A. Alignment of human and mouse CCL20 promoters. Position +1 indicate initiation site for transcription and first codon is highlighted in grey. * indicates homology in the sequence alignment. Critical features and transcription factor binding sites are underlined. B. Schematic representation of the different reporter constructs. (*luc*: firefly luciferase gene, pA: polyadenylation signal). “IRES” stands for internal ribosome entry site. The cDNA *eyfp* encodes the enhanced yellow fluorescent protein. Positions related to promoter are coincident with panel A. C. Functional analysis of *Ccl20-luc in vivo*. BALB/c mice were transfected for 48 h by hydrodynamic shock with *Ccl20-luc* and TK-renilla plasmids, treated next i.v. for 8 h (5 µg flagellin or PBS), and liver luciferase activity was determined. Results are expressed as relative luciferase activity using PBS-treated mice as reference and renilla activity as normalizer for transfection efficiency. Data are given as mean ± SD (n = 8; * p<0.05 Mann-Whithney test) and are representative of two experiments. D. NF-κB site but not Ets1 site is required for the activation of transcriptional fusion by flagellin. Experiments were conducted as described in C using different luciferase reporter *Ccl20-luc*_[−299–+6]_ plasmids harboring deletions or mutations as shown in B. E. Liver response induced by flagellin depends on TLR5. Experiments were conducted as described in C using *Ccl20-luc*, TK-renilla and TLR5shRNA or control scramble plasmid. In addition to flagellin, a group of animals was treated i.v. with 5 µg LPS. Data are given as mean ± SD (n = 6–8; * p<0.05 n.s.: non significant differences, Mann-Whithney test) and are representative of three experiments. F. Liver response induced by flagellin is TLR5 dependent. Experiments were conducted as described in C using *Ccl20-luc*, TK-renilla and dominant negative TLR5_393stop_ or control pcDNA3 plasmids. Data are given as mean ± SD (n = 6–8; * p<0.05 n.s.: non significant differences, Mann-Whithney test) and are representative of two experiments. G. Functional analysis of the *Ccl20-luc-IRES-eyfp* transcriptional fusion to be used for transgenic mice generation. Experiments were conducted as described in C using *Ccl20-luc-IRES-eyfp*. Data are given as mean ± SD (n = 8; * p<0.05 Mann-Whithney test) and are representative of two experiments.

The *Ccl20* gene (about 1.4 kb of promoter and 1.0 Kb of transcribed gene) was originally amplified using the following primers: *CTCGAG* CACAATGGAGAGGAATGACCA (*XhoI*) and *AAGCTT* ATGTACGAGAGGCAACAGTCG (*HindIII*). The promoter was then cloned using *XhoI* and the endogenous *KpnI* site close to the start of transcription (Figure 1A) into the firefly luciferase reporter pGL-3 basic vector (Promega, USA). The resulting construct p*Ccl20-luc* (−1377/+11) harbors 1388 bp (1377 bp upstream transcription start site and 11 bp of transcribed region) of the *Ccl20* promoter region. To generate the transgenic animals, an IRES-EYFP cassette obtained from Dr. Richard M. Locksley [31] was cloned downstream the *luc* cDNA of p*Ccl20-luc* to give rise to the plasmid p*Ccl20-luc-IRES-eyfp*. We also used the reporter plasmids harboring *Ccl20* promoters −299/+6, −85/+6, −299/+6 (NF-κB mutant) and *luc* fusions previously described by Fujiie et al. [15]. An Ets-1 mutant fusion was constructed using the −299/+6 reporter fusion as template to change the (−156) GCAGGAAGT (−147) Ets-1 site in GCATTCAGT. To define the TLR5 specificity of flagellin response, two sets of constructs were used. First, plasmids expressing functional shRNA under the control of 7SK promoter targeting Tlr5 or coding for a scrambled shRNA sequence (Invivogen, France). Second, the cDNA coding for the recombinant mouse TLR5 (residues 1 to 392 with codon 393 changed in a stop codon), a sequence equivalent to the dominant negative human TLR5 [25] was cloned into the *HindIII* and *NotI* sites of the vector pcDNA3 using the PCR fragment obtained with primers: *AAGCTT* GGATCATGGCATGTCAACTTGACTTGC and *GCGGCCGC* TCAGAGATCTAAGGTTTGTAACG. All plasmids were verified by re-sequencing.

### Hydrodynamic Shock Injections

BALB/c mice were injected with combination of plasmids diluted in 1.4 mL Ringer solution (147 mM NaCl, 4 mM KCl, 1.13 mM CaCl_2_, pH 7.5) via tail vein as described previously [32]. Typically, 25 µg Firefly luciferase reporter plasmid (*Ccl20* promoter) and 2.5 µg Renilla luciferase plasmid pRL-TK (Promega) for the normalization of transfection efficiency ±25 µg plasmid for interference or dominant negative or control plasmids, were used for each animal. Plasmid injection was completed in less than 5 s and the level of activation of reporter plasmids was assessed 48 h later. For this purpose, animals were injected i.v. with 200 µl PBS ±5 µg flagellin as described previously [19], [21]. Different organs including the liver, spleen, or lung were sampled 6 h later to measure luciferase activity (Firefly and Renilla), using Dual-Luciferase Assay (Promega), following manufacturer instructions.

### Generation and Analysis of Transgenic Animals

Transgenic animals were generated on a C57BL/6J genetic background at the Institut Clinique de la Souris in Strasbourg, France. Briefly, the plasmid p*Ccl20-luc-IRES-eyfp* was purified and injected in fertilized eggs from C57BL/6J. Several offspring founders were generated and identified by PCR genotyping and bred with C57BL/6J to produce F1. The first screening to identify interesting reporter animals was performed on the F2 generation by administration of flagellin and measurement of luciferase activity in differen organs (see below). Classical genotyping of mice was performed on tail genomic DNA using the following set of primers for control (Cpxm1-F: ACTGGGATCTTCGAACTCTTTGGAC and Cpxm1-R: GATGTTGGGGCACTGCTCATTCACC), 5′-amplification of construct (D-F: GTGACAGGATGAGGCAGATTAATCAATGGG and D-R: CTTTACCAACAGTACCGGAATGCCAAG), and 3′-amplification of construct (F-F: GCCCAGAAGGTACCCCATTGTATG and F-R: GTTTACGTCGCCGTCCAGCTC). In some assays, genomic DNA was assessed using real time quantitative PCR with the following primers specific for *luc* (LUC-F: TGAGTACTTCGAAATGTCCGTTC and LUC-R: GTATTCAGCCCATATCGTTTCAT) and Actb (Actb-F: CGTCATCCATGGCGAACTG and Actb-R: GCTTCTTTGCAGCTCCTTCGT). Since *Ccl20-luc-IRES-eyfp* expression was stable over F1 to F8 (not shown), one can consider that the germline transmission of transgene is effective and not subjected to attenuation of expression.

Transgenic animals were stimulated with heat-inactivated Salmonella *enterica* (10^8^ CFU) or flagellin from Salmonella *typhimurium* (5 µg/animal) (FLA-ST, InvivoGen, San Diego, CA, USA) via i.p. or i.v routes for 4 h or as indicated.

### Luciferase Assays

At indicated times, mice were sacrificed by cervical dislocation or pentobarbital injection and samples of liver, spleen, lung and small intestine were homogenized in lysis buffer (Luciferase Assay System, Promega, MA, USA) using an ultra-turrax equipment (T10 basic, IKA, Staufen, Germany). Luciferase activity was determined using Luciferase Assay System by measuring the bioluminescence emission in plate luminometer (LUMIstar OPTIMA, BMG labtech, Ortenberg, Germany) according to manufacturer’s instructions.

### Gene Expression Analysis

After stimulation, animals were sacrificed, organs recovered and homogenized in RA1 lysis buffer (GE Healthcare, Pittsburgh, USA) and total RNA was extracted with Nucleo Spin RNA II kit (Macherey-Nagel, Düren, Germany). Reverse transcription was performed using 200 U M-MLV reverse transcriptase (Invitrogen, USA), 0.5 mM dNTPs, 1 nM random primers, 20 U RNAseOUT, 7.5 mM DTT, 1X reaction buffer and 400 ng of RNA in a final volume of 20 µl. The RT reaction was performed at 25°C for 10 minutes followed by 37°C for 60 minutes. For real-time PCR, MyiQ Single color Real-time PCR Detection System (BioRad, Hercules, CA, USA) and SYBR® Green Master Mix (Invitrogen) were used. A standard protocol of 40 cycles of denaturation 15 sec at 90°C and annealing/extension 1 min 60°C amplification was used. Primers for pro-inflammatory CCL20 and mouse β-actin were previously designed and described [20], [33]. Relative mRNA expression of each chemokine was evaluated through the 2-ΔΔCt method and the β-actin gene was used as normalizer gene [20].

### *In vivo* Imaging Experiments

A comparative analysis of the effect of the two agonists of the innate immunity described before was done when administered via i.p. and i.v. in *CCL20-luc-IRES-eyfp* and *NFκB-luc* control mice of 8 to 12 weeks of age. Activity of the reporter gene was studied in different organs by *in vivo* imaging at 4, 8, 12 and 24 h pos-stimulation, through injection of 150 mg/kg of luminogenic substrate for luciferase (D-Luciferin Firefly, Caliper Life Sciences, MA, USA), keeping the animal anesthetized with isofluorane (Isoforine, Cristalia, SP, Brazil) for 5 min in *in vivo* imaging analysis system (MS FX PRO, Carestream Health Inc, USA). Intensity of innate response activation in different organs was evaluated using the software Carestream molecular imaging v 5.3.3.17476.

For *Ccl20-luc* reporter mice, an imaging capture protocol of 5 min exposure was established, in comparison of a shorter protocol used for the NF-κB model. In both cases, a previous X-ray exposure followed by the bioluminescence filter was performed. Images obtained from each animal were regionalized (ROI) in three for *Ccl20-luc-IRES-eyfp* mice: thorax, upper abdomen and middle abdomen, and in five for NF-κB mice: head and neck, thorax, upper abdomen, middle abdomen and lower abdomen. For each ROI the software calculated net and mean intensity of photon emission per mm^2^, plotting average ± SE for each treatment and acquisition time.

## Results

### Construction and Characterization of Mouse *Ccl20* Reporter Fusions

With the aim to generate a reporter mouse strain, the *Ccl20* gene promoter was linked to the Firefly luciferase gene *luc*, giving rise to a transcriptional fusion (Figure 1). The 1388 bp fragment that was used as the *Ccl20* promoter contains 1377 bp upstream the initiation of transcription site of murine *Ccl20* gene as defined previously by Fujiie *et al.* [15] (Figure 1A–B). By transient transfection on human epithelial cell lines Caco-2 and HEK293, functional activity of reporter upon pro-inflammatory stimulation by the TLR5 agonist flagellin was confirmed (data not shown). The activity of the reporter fusion was next assessed *in vivo* using hydrodynamic shock injection followed by stimulation with flagellin (Figure 1C). This approach allowed a transient transfection of mouse liver cells with the plasmid(s) injected by the i.v. route. The flagellin stimulation was able to induce >100-fold the luciferase activity in liver in contrast to PBS-stimulation of transfected animals. These results are in accordance with previous reports indicating that flagellin increases liver pro-inflammatory transcription [29].

Additional experiments were performed to further characterize the activity of the reporter in response to flagellin stimulation. First, we defined that NF-κB site is essential to the activity of the *Ccl20* promoter using the constructs of Fujiie *et al.* [15]. Indeed, reporter fusions deleted (−85/+6) or mutated within the NF-κB site were totally abrogated in the capacity to respond to flagellin (Figure 1D). We also generated a fusion harboring a mutation in the Ets-1 site (−156/−147). This mutation did not affect the response to flagellin, thereby indicating that the liver response is dominated by pro-inflammatory NF-κB signaling (Figure 1D). Next, we assessed the role of direct TLR5 stimulation on liver luciferase activity (Figure 1E–F). To this aim, *Tlr5*-specific shRNA encoding plasmids were co-administered with the *Ccl20-luc* reporter construction by hydrodynamic shock and mice were stimulated with flagellin. Under these conditions, impairment of liver TLR5 expression abrogated completely the reporter expression in response to flagellin (p<0.001) but not LPS (Figure 1E). In contrast, the control (scramble) shRNA plasmid still displayed inducible luciferase activity independently of stimulation. Finally, the requirement of TLR5 signaling was studied using the mouse counterpart of human dominant negative TLR5, i.e. mTLR5_393stop_. Injection of the plasmid encoding mTLR5_393stop_ was associated to >90% decrease response to flagellin (Figure 1F). In conclusion, the direct flagellin-TLR5 interaction on liver cells seems to be the major pathway that control *Ccl20* early expression upon systemic flagellin delivery.

### Generation and Functional Analysis of *Ccl20* Reporter Mice

**Figure 2:**
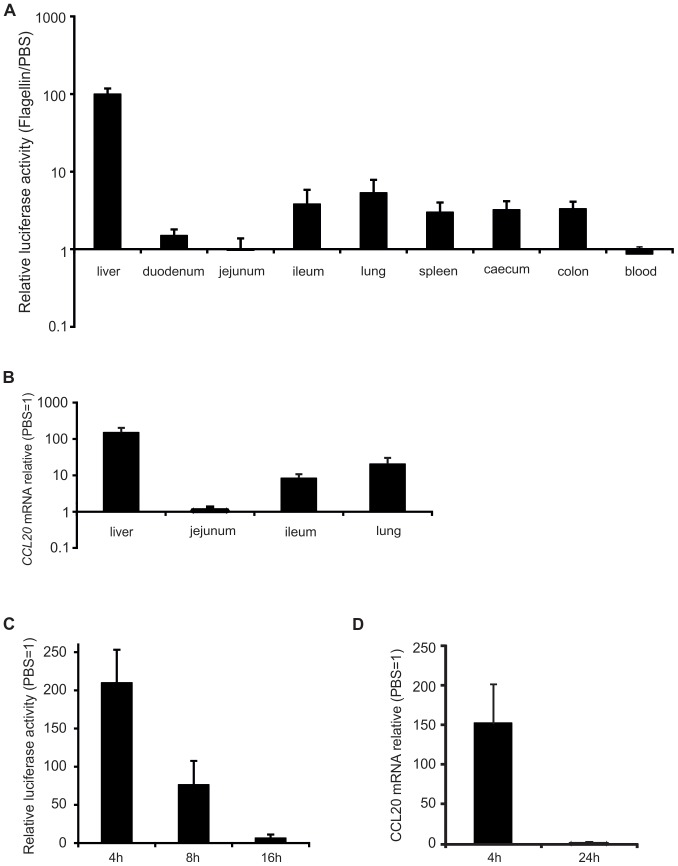
Characterization of *Ccl20-luc-IRES-eyfp* transgenic line. Transgenic mice were treated with 5 µg flagellin or PBS i.v. and were sacrified at indicated times. Organs were recovered for determination of luciferase activity or *Ccl20* expression. A. Analysis of fusion expression in several tissues after flagellin stimulation. Luciferase activity was measured 4 h post-treatment. Results are expressed as relative luciferase activity using PBS-treated mice as reference. Data (n = 8) are given as mean ± SD and are representative of two experiments. B. Flagellin-specific upregulation of *Ccl20* transcription in tissues. *Ccl20* mRNA levels were measured in different organs by RT-qPCR 4 h post-treatment. Results are expressed as relative mRNA levels using to PBS treated mice as reference. Data (n = 5) are given as mean ± SD and are representative of two experiments. C. Kinetics of liver activation by flagellin as measured by fusion expression. Luciferase activity was measured in liver at different times after treatment. Results (n = 7) are expressed as in A. D. Kinetics of liver activation by flagellin as measured by mRNA levels. *Ccl20* mRNA level measured by RT-qPCR at 4 h and 24 h. Results (n = 5) are expressed as in C.

The p*Ccl20-luc* plasmid was further engineered to incorporate the *IRES-eyfp* cassette downstream of *luc* cDNA. The resulting plasmid p*Ccl20-luc-IRES-eyfp* was found to respond as effectively as p*Ccl20-luc* in the hydrodynamic shock model (Figure 1G). However, we were not able to detect increase of EYFP fluorescence in liver of flagellin-stimulated mice (data not shown). The generation of a stable reporter mouse strain by transgenesis was then started using the p*Ccl20-luc-IRES-eyfp* DNA at the Institut Clinique de la Souris. Among several PCR positive founders, 5 clones were selected for expansion as F1 generation and characterization. The F2 mice derived from three selected F1 founders (clones 61, 68 and 72) were analyzed for the relative levels of luciferase activity in various organs upon stimulation with flagellin (Figure S1). The animals derived from clone 61 were selected for further expansion and analysis. In several tissues of *Ccl20-luc* mice derived from clone 61, the luciferase activity was low or close to background signals at the steady state or after mock treatment (data not shown). In contrast, the flagellin treatment was consistently able to induce the expression of reporter fusion as detected by the increase of the luciferase activity (Figure 2). The maximal activation was associated with the liver although significant luciferase activity was observed in lung, spleen, ileum and colon (Figure 2A). However, *Ccl20* reporter fusion expression was almost undetectable in blood, duodenum and jejunum. These results highly correlated with the levels of *Ccl20* transcripts (Figure 2B). Indeed, liver *Ccl20* mRNA levels were increased 140±30 fold upon flagellin stimulation, whereas the increase was 30±8 and 8±3 fold in lung and ileum, respectively. The fusion expression in liver was also assessed at different times after flagellin stimulation. We observed a fast induction of luciferase activity (200±50 fold) 4 h post-treatment associated to a gradual decrease at later time points (Figure 2C). This rapid activation correlates with a rise in *Ccl20* mRNA levels of around 150 fold at 4 h post-treatment that return to basal levels at 24 h (Figure 2D). Interestingly, qPCR analysis of genomic DNA derived from F1 to F7 generations revealed that the transgene copy number is stable as defined by relative levels of genomic DNA specific for luciferase and beta actin genes (data not shown). Altogether, our data show that the *Ccl20-luc* reporter mice represent an appropriate model to monitor in real time the activity of *Ccl20* promoter.

**Figure 3:**
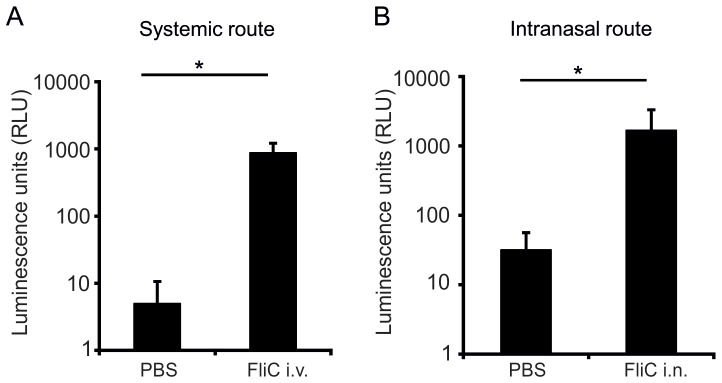
Luciferase activity is induced by treatment with flagellin by different routes. *Ccl20-luc-IRES-eyfp* mice were treated with flagellin (5 µg) or PBS by i.v. (A) or i.n. (B) route. Lungs were sampled 4 h later and luciferase activity was measured. Results (n = 7) are expressed as mean ± SD (* p<0.05 Mann-Whithney test) and are representative of two experiments.

As CCL20 can be induced at various mucosal sites in response to pro-inflammatory stimuli [14], the activation of the reporter in lung mucosa after intranasal and systemic flagellin administration was compared. A significant induction of the luciferase activity was observed in both cases (Figure 3A).

### Real Time Bio-imaging of *Ccl20* Promoter Activity

**Figure 4:**
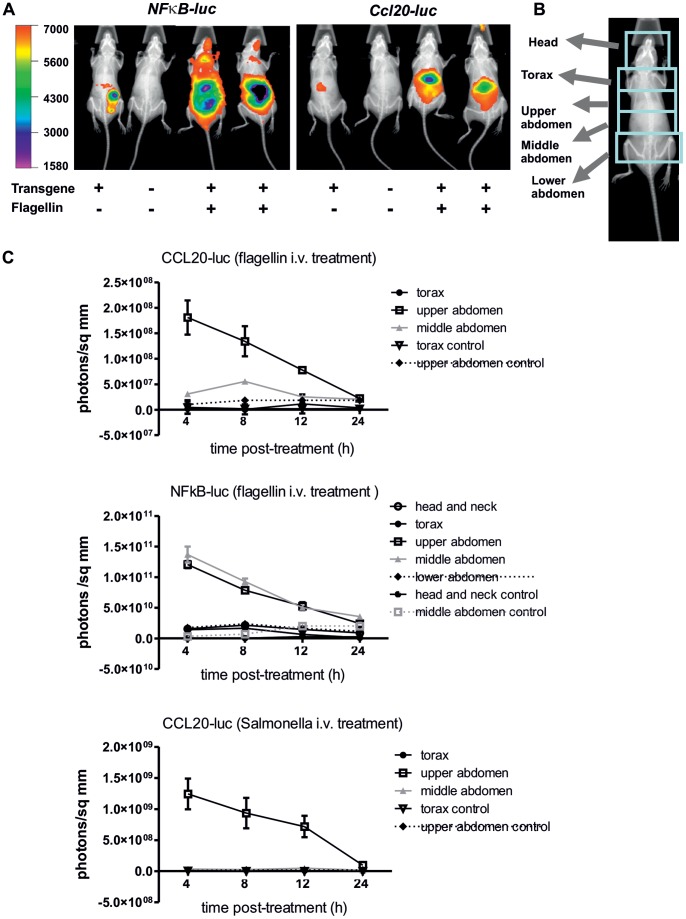
*In vivo* imaging shows specific liver response to inflammatory stimuli. A. *NFκB-luc* and *Ccl20-luc-IRES-eyfp* mice imaging 4 h after flagellin (10 µg) i.v. treatment. Image acquisition was performed as indicated in methods section and was stared 1 minute after administration of luciferin. X-ray and luminescence images are superimposed by the software. Luciferase activity is depicted in a pseudocolored scale, as indicated. Non transgenic and transgenic-PBS treated mice were used as controls. B. Animal body was arbitrarily divided in different anatomic regions, using the X-ray image as reference. C. Kinetic analysis of *NF-κB-luc* and *Ccl20-luc-IRES-eyfp* mice stimulated i.v. with flagellin (10 µg) or heat-killed Salmonella (10^8^ CFU equivalent). Absolute photon emission was calculated for anatomical regions defined in B. For each acquisition, luciferin was injected 1 minute before the measurement as stated in A. Results corresponding to each anatomic region are depicted with different symbols, as indicated in each graph. Mean values and SD for each group are depicted (n = 4).

A major use of luciferase reporter fusion is the real time monitoring of gene expression by bio-imaging [34]. The *Ccl20-luc* reporter mouse was used for *in vivo* imaging studies at different times after systemic stimulation, i.e. i.v./i.p. administration of flagellin or heat-inactivated *Salmonella* (Figure 4). The *NF-κB-luc* reporter mice strain that harbors artificial promoter with tandem NF-κB motifs was used as control to evaluate the intensity of the pro-inflammatory response [35]. In 4 h-treated *NF-κB-luc* mice, flagellin induced a strong luciferase activity in the abdominal region (liver and gut) and the respiratory tract compared to untreated mice (Figure 4A). In the same conditions, luciferase activity was detected in *Ccl20-luc-IRES-eyfp* mice but was restricted to the abdomen. The system used for *in vivo* imaging allows the quantification of the luminescence emission in defined areas. Different anatomic regions were defined to get a comparative analysis of regionalized activation (Figure 4B). The kinetic analysis performed in *Ccl20-luc-IRES-eyfp* mice showed that upon flagellin stimulation, the maximal activity was mainly limited to upper abdomen, with a peak at 4 h and a progressive decrease to basal levels at 24 h. In the middle abdomen, a lower luminescence and a different kinetic (maximum at 8 h) was observed (Figure 4C). The magnitude of luciferase activity was more important in *NFκB-luc* mice than in *Ccl20-luc-IRES-eyfp* mice, with significant differences in kinetics and anatomical expression. When *Ccl20-luc-IRES-eyfp* mice were stimulated with heat-inactivated *Salmonella*, luciferase expression patterns were found to be similar to flagellin-induced treatment although the global magnitude was higher (Figure 4C and data not shown). Altogether these results showed that the *Ccl20-luc-IRES-eyfp* mice could be used for *in vivo* real time imaging. Furthermore, the *Ccl20-luc-IRES-eyfp* mice showed a specific pattern of expression to systemic challenge charcterized by specific activation in the liver.

## Discussion

In the present work we have characterized some features of the *Ccl20* promoter and generated the transgenic reporter mouse line *Ccl20-luc-IRES-eyfp* that recapitulates the transcriptional activity of *Ccl20* in various organs. We have shown that the transgenic mouse line can be used for *in vivo* imaging of CCL20 response, representing a novel tool for the examination of physiopathological situations that may include the CCL20-CCR6 axis as a major component.

From the initial descriptions of mouse *Ccl20* and human *CCL20* promoters, the conservation of several features was appreciated. First, the proximal NF-κB binding site was found to be critical to the promoter activity in steady state (constitutive) and pathological (inducible) conditions [15], [16], [36], [37]. We have previously shown that the NF-κB binding site can bind different NF-κB combinations [19]. When the canonical p65/p50 pro-inflammatory NF-κB dimer binds the site, a highly inducible CCL20 response is triggered as a feature of pro-inflammatory stimuli. Besides, the same site can bind RelB/p52 NF-κB that is involved in the NF-κB alternative pathway function, thereby promoting a sustained transcription of CCL20 encoding gene after engagement of several TNFR superfamily members such as LTβR or TWEAKR [20], [38]. This pathway has been involved in the constitutive expression of CCL20, particularly in sites such as the follicle associated epithelium in mucosal sites. By targeted mutations, Kwon et al. showed that ETS binding sites in the proximal promoter region were critical for *Ccl20* expression in intestinal epithelium [39]. In this work, we have shown that in the mouse promoter the NF-κB binding site is critical for *in vivo* liver inducible activation; however the ETS site positioned in −156/−147 is dispensable to this activity. Among the various ETS transcription factors, the intestinal epithelium specific ESE-1 transcription factor was shown to bind the *Ccl20* promoter [39] and contribute to the inducible expression in intestinal epithelium [20]. The high inducible activity observed in liver may be contributed by other specific tissue transcription factors that are not acting on ESE-1 binding site and have not yet been identified.

In concordance with previous reports [37], [40], we have observed that *Ccl20* expression upon different pro-inflammatory stimuli is maximal in liver. LPS, as well as proinflammatory cytokines such as TNFα, are potent inducers of liver *Ccl20* expression [37], [41]. Since flagellin stimulation may trigger the production of TNFα by myeloid cells [42], it may be expected that by systemic administration of flagellin, CCL20 expression can be contributed initially by direct PRR-PAMP interaction and secondary by indirect activation by cytokines such as TNF that are secreted upon PAMP stimulation. However, we have shown that impairing TLR5 activity in liver *in vivo* abrogates completely the CCL20 liver response, indicating that direct PRR-PAMP interaction is the main trigger of *Ccl20* liver expression in this case (Figure 1). The kinetics of *Ccl20-luc* expression observed *in vivo* upon flagellin or heat-killed salmonella stimulation supports this hypothesis (Figures 2 and 4).

We have shown that the transgenic reporter mice are suitable for *in vivo* imaging experiments. The capacity of doing a whole body scanning for luciferase activity allows determining that upon proinflammatory stimulation, CCL20 production is maximal in the liver, in coincidence to what was shown by determination of enzymatic activity and mRNA in a case by case approach (Figure 2). Other organs that were not included in the screening such as skin or thymus were not detected as major sites of CCL20 induction in the *in vivo* imaging analysis. It must be noted that there are several limitations to this approach. The luciferase derived green bioluminescence has limited capacity to pass though tissue, so emission by internal organs should be over a certain threshold to be detected by the CCD camera used for the acquisition [43]. This threshold may not be similar for different body regions, since their proximity to body surface is not necessary the same. These differences may explain why the luciferase activity detected in lungs upon flagellin treatment was evident when analyzed on a lung lysate; however the light emission in thorax detected by *in vivo* imaging was almost similar to background emission.

The *in vivo* imaging approach allowed us to perform a simple comparison of activity against the NF-κB-luciferase reporter mouse strain that harbors an artificial promoter containing tandem NF-κB binding sites [35] and was widely used to monitor *in vivo* NF-κB activation [44]. Although CCL20 expression is highly dependent on NF-κB activation, the luciferase activity observed was not equivalent in *Ccl20-luc* and *NFκB-luc* mice. For instance, *NFκB-luc* mice showed high luciferase activity at steady state and widespread tissue expression upon pro-inflammatory stimulation compared to *Ccl20-luc-IRES-eyfp* mice. The differences observed between the two reporter mice strains reflect the activity of their respective promoters. The native mouse *Ccl20* promoter contains several regulatory regions that respond to several transcriptional modulators including NF-κB. As discussed previously, specific combinations of transcription factors are required to activate *Ccl20* expression with respects to nature of tissue/cell and environmental signals.

We have shown that the *Ccl20-luc-IRES-eyfp* reporter mice recapitulate CCL20 expression in tissue- and stimuli-specific manner. This mouse model may constitute an important tool for research on liver inflammation and on any diseases where CCL20 may constitute an important element of the pathophysiology, such as NASH [45] autoimmune hepatitis [46] sterile inflammation and atherosclerosis [47] or ischemia reperfusion injury [48].

## Supporting Information

Figure S1 **Functional screening on several** ***Ccl20-luc-IRES-eyfp*** **mice lines originated from different founders.** *Ccl20-luc-IRES-eyfp* mice from founders #61, #68 and #72 were treated i.p. with 5 µg flagellin or PBS. Tissues were sampled 4 h post-treatment and luciferase activity was determined on tissue lysates. Results are expressed as mean luminescence (n = 5–7).
(TIF)
